# Does our brain use the same policy for interacting with people and manipulating different objects?

**DOI:** 10.3389/fncom.2014.00170

**Published:** 2015-01-05

**Authors:** Fatemeh Yavari

**Affiliations:** Biomedical Engineering Department, Amirkabir University of TechnologyTehran, Iran

**Keywords:** internal forward model, internal inverse model, Modular organization, Schematic processing, Stereotypes

## Introduction

Our first impression of other people is greatly affected by our previous experiences. Schematic processing, proposed in social psychology, explains our behavior in interacting with other people. It suggests existence of different schemas in our brain for different groups of people, e.g., extroverts, introverts, shy, women, men, etc. and also schemas related to special people like our parents, close friends, supervisor, and even ourselves. Each schema is recalled when we meet the corresponding person/personality (Atkinson, 1996).

On the other hand there is a relatively well accepted theory–model based theory- in motor control and learning studies (Daw and Dayan, 2014; Dayan and Berridge, 2014). It suggests existence of some internal models (forward and/or inverse) in the brain which help us for planning and execution of the actions.

Although these two viewpoints may seem very distinct, there are some interesting similarities between them, which are explained in the following section. I hypothesize that these correspondences may suggest that the brain employs same algorithms in dealing with both situations.

Understanding the brain function is a great challenge for many scientists. Further evaluation of the proposed hypothesis may be helpful to achieve better understanding of the brain function, as advances in each field may encourage new ideas in the other one.

In the following sections each of the two viewpoints and then their similarities are explained.

## Stereotypes in social psychology

Stereotype is defined as “a fixed, often simplistic generalization about a particular group or class of people (Cardwell, 2014)”. Stereotypes, schemas, and schematic processing enable us to efficiently organize and process the huge volume of input information to our brain. Instead of processing every little detail about a new person, we can just recall the most similar schemas and generally categorize the person e.g., based on his most obvious physical features (Atkinson, 1996). Stereotypes enable us to respond rapidly in situations which we have had similar experience. Despite all the benefits, stereotypes may also result in prejudice. Since they bias our impressions, they can have very negative and even mortal (e.g., Amadou Diallo case) consequences (Atkinson, 1996).

## Internal models in motor control and learning studies

Internal models are defined as representations of external objects and/or our body organs in the brain (Kawato, 1999) (see Yavari et al., 2013 for a review). They are categorized into “forward” and “inverse” which mimic the “input-output” and “output-input” relationship of the related object/organ, respectively. Model-based theory suggests that motor learning/adaptation leads to formation/modification of internal models (Hunter et al., 2009). Kawato et al. have proposed co-existence of multiple pairs of internal forward-inverse models in the brain and therefore, a modular structure for motor control and learning (Wolpert and Kawato, 1998; Haruno et al., 2001, 2013; Doya et al., 2002; Imamizu et al., 2003; Wada et al., 2003). Based on this idea, which has been supported by different behavioral and imaging evidence (Wolpert and Kawato, 1998; Imamizu et al., 2003), there are an inverse (controller) and a forward (predictor) internal model within each pair. Contribution of each controller to the final motor command is determined based on accuracy of the linked forward model. This modular structure can explain our remarkable ability in motor learning, adaptation, and behavioral switching (Haruno et al., 2013).

## Similarities of the two mentioned viewpoints

Some similarities between the two mentioned viewpoints are described here:

- Both processes are implicit and unconscious. Associations which are activated through stereotypes can be deeply learned and become automatic (as shown by priming-based experiments) (Rudman and Borgida, 1995; Atkinson, 1996; Bargh et al., 1996). Similarly, after enough practice, a motor skill (such as driving) can be performed unconsciously and without need to attention (Schmahmann, 1997).
- Based on primary effect, the initial information which we receive (e.g., hear) about a person significantly bias our impression of him/her. This effect has been explained using schematic processing as follows: we try to achieve a general impression about the person by searching for the most consistent schema or stereotype with the input information. This schema determines our judgment about his/her personality.
  There is a same process about internal models in motor control: when manipulating a new tool the most suitable FM/IM pair is activated based on context, e.g., by looking at the object's appearance, and the corresponding IM is used as controller. In the next trial, the pair which produced the least minimum prediction error will be activated and used (Wolpert et al., 2003).
- Stereotypes help us in inference, i.e., making judgment beyond the given information. For instance when we hear that someone is affectionate, we will probably consider him/her also a generous person (Atkinson, 1996). There is conceptually similar to generalization in motor learning which has been proposed to be resulted from internal models. An internal model formed by practicing a motor action under a special condition can partly be generalized to other circumstances. For example, practicing a movement with the right hand generates a learning which partly generalizes to the left hand (Sainburg and Wang, 2002; Wang and Sainburg, 2006; Balitsky Thompson and Henriques, 2010).
- One of the famous models in impression formation is the continuum model (Fiske et al., 1999) which describes the whole range of processes from stereotypes to individuation. Based on this model, automatic stereotypes are the first psychological process activated when we meet someone for the first time. We categorize this person unconsciously and automatically in terms of age, sex, and ethnicity. This is called initial categorization. If the person is important for us, we obtain more information about him (piecemeal integration) and finally judge him based on his individual characteristics (individuation). Proceeding from stereotypes toward individuation happens slowly (Atkinson, 1996).
  Based on internal model theory learning a new motor skill goes through an almost similar process: When we try to manipulate a new object, in the early stage, CNS combines output signals from internal models of most similar (and familiar) objects. After some practice we learn to manipulate the new object skillfully and the reason is the special internal model which has been formed for it (Imamizu and Kawato, 2012). Depending on the complexity of the new motor task, its learning would need different time. It could take even years (e.g., for professional athletes).

As it can be seen in both situations, in a new condition reliance is more on previous experience, while gathering more information over time leads to formation of special new internal model/stereotype.

## Conclusion

Human brain is probably the most fascinating creation in the world. Many scientists in different fields are trying to understand its function. Here I hypothesized that maybe our brain applies the same policy for some distinct applications, e.g., social interaction and manipulating different objects.

It worth mentioning that internal models have been proposed not only in motor control and learning, but also in some other fields such as control of mental activities (Ito, 2008), cognitive planning (Dayan and Yu, 2006), and decision making (Daw et al., 2011). These processes may even have more in common with stereotypes.

It would be interesting to also compare the corresponding neural substrates for stereotypes and internal models. Cell recording in some animal studies (Liu et al., 2003; Cerminara et al., 2009; Laurens et al., 2013) and also imaging studies (Imamizu et al., 2000, 2003; Blakemore et al., 2001; Kawato et al., 2003; Higuchi et al., 2007; Milner et al., 2007) suggest lateral and anterior cerebellum as the probable site of formation or storage of internal models. Some studies have suggested that motor cortex and other frontal motor areas have important roles in computation of internal models (Li et al., 2001; Shadmehr, 2004; Richardson et al., 2006; Shadmehr and Krakauer, 2008). Medial prefrontal cortex (mPFC) has been proposed as a candidate region in model-based evaluation (Hampton et al., 2006, 2008; Valentin et al., 2007; Daw et al., 2011). On the other hand, some neuroimaging studies have shown mPFC as a crucial region in social inferences, (Mitchell et al., 2005a,b, 2006), and judgments of warmth and competence (Harris and Fiske, 2006). Activity in middle mPFC is shown to be associated with thinking about either the self or a similar other (Ida Gobbini et al., 2004; Mitchell et al., 2006); while activity in dorsal mPFC is associated with thinking about a dissimilar other. Therefore, mPFC seems to be important for ingroup and outgroup perception (Amodio and Lieberman, 2009). Perceiving a person as a social being, which has been proposed to form the basis of prejudice (Qiu, 2006), has been suggested to be dependent on dorsal mPFC (Amodio and Lieberman, 2009). Therefore, PFC seems to be a crucial brain region for both internal models and stereotypes.

Further evaluation of the proposed hypothesis may be helpful to achieve better understanding of the brain function. For example as it was mentioned, stereotypes have significant effect on our social life and undeniable effect on impression formation. They sometimes have negative (even mortal) impact on our judgments, because they bias our impressions. The more we increase our knowledge about this concept, the more we can modify our thoughts in a good manner.

Discoveries in each field may lead to new findings in the other. For instance it has been shown that stereotypes may be activated through unconscious priming; e.g., in an experiment by Bargh et al. (1996) seeing images of young African American men triggered more aggressive behavior compared to images of young Caucasian men, even though the images were displayed for less than thirty thousandths seconds (subliminally) (Atkinson, 1996). This observation can be verified about motor actions as well. For example to investigate if seeing a special tool, such as a piano, can prime the piano playing skill. This can be both useful for better understanding the motor related mechanisms in the brain and also in practical applications such as preparing the athletes before their match to achieve better results.

The proposed hypothesis needs to be verified by some specially-designed experiments.

### Conflict of interest statement

The author declares that the research was conducted in the absence of any commercial or financial relationships that could be construed as a potential conflict of interest.
